# Boston School of Medical Specialties

**Published:** 1867-11

**Authors:** 


### Boston School of Medical Specialties.

The following physicians have associated themselves for the purpose of giving instruction in their respective departments to advanced students, and to physicians who may be desirous of fitting themselves for special practice. It is thought, also, that there are some general practitioners who will be glad to refresh their memories.

This seems the first effort of the sort in this country, and is undertaken by men very widely and favorably known in their respective departments.

HENRY G. CLARK, M. D., (Bowdoin College) Surgeon to the Massachusetts General Hospital. Lecturer on Public Hygiene and Medical Jurisprudence.

HORATIO R. STORER, M. D., (Harvard University) Pupil of Simpson, of Edinburgh, and Professor in Berkshire Medical College. Lecturer on the Diseases of Women.

Prof. Storer's course, the present winter, will be upon Uterine Diagnosis; and will be exclusive of that upon the Surgical Diseases of Women, to be delivered in December.

GUSTAVUS HAY, M. D., (Harvard University) Pupil of Arlt, of Vienna, and Surgeon to the Eye and Ear Infirmary. Lecturer on Operative Ophthalmology.

B. JOY JEFFRIES, M. D., (Harvard University) Pupil of Hebra, of Vienna; Lecturer in Berkshire Medical College, Surgeon to the Eye and Ear Infirmary, and Physician to the Boston Dispensary. Lecturer on the Diseases of the Skin.

JOSIAH H. STICKNEY, M. D., F. R. C. V. S., (Harvard University) Pupil of Varnell, of London. Lecturer on Veterinary Medicine and Surgery.

THOMAS B. HITCHCOCK, M. D., (Harvard University) Lecturer on the Diseases of the Teeth.

FRANCIS C. ROPES, M. D., F. R. C. S. E., (Harvard University) Pupil of Langenbeck, of Berlin; Physician to the Boston Dispensary, and Surgeon to Out Patients at the City Hospital. Lecturer on Surgical Deformities.

SAMUEL W. LANGMAID, M. D., (Harvard University) Pupil of Mackenzie, of London, and Physician to the Boston Dispensary. Lecturer on Diseases of the Throat.

DAVID L. LINCOLN, M. D., (Harvard University) Pupil of Widerhofer, of Vienna, and District Physician to the Boston Dispensary. Lecturer on the Diseases of Infants and Children.

JOSEPH G. PINKHAM, M. D., (Long Island College Hospital) Pupil of Bloxam, of London, and Professor of Chemistry in Berkshire Medical College. Lecturer n Toxicology.

FRANCIS B. GREENOUGH, M. D., (Harvard University) Pupil of Zeissl, of Vienna. Lecturer on Venereal Diseases.

GEORGE F. H. MARKOE, Member of the American Pharmaceutical Association. Lecturer on Practical Pharmacy.

